# The NFAT5–AR Axis Is Associated with Hyperosmolarity, Renal Dysfunction, and Neutrophil-Related Inflammatory Markers in Diabetic Retinopathy

**DOI:** 10.3390/ijms27021102

**Published:** 2026-01-22

**Authors:** Fátima Sofía Magaña-Guerrero, Beatriz Buentello-Volante, Norma Angélica Magaña-Guerrero, Óscar Vivanco-Rojas, Alfredo Domínguez-López, Yonathan Garfias

**Affiliations:** 1 Cell and Tissue Biology Department, Research Unit, Institute of Ophthalmology Conde de Valenciana Foundation, Cimpalpopoca 14, Mexico City 06800, Mexicobbuentello@institutodeoftalmologia.org (B.B.-V.); namg6398@gmail.com (N.A.M.-G.); oscarv@bq.unam.mx (Ó.V.-R.); adominguezl@facmed.unam.mx (A.D.-L.); 2 Biochemistry Department, Faculty of Medicine, Universidad Nacional Autónoma de México, Av. Universidad 3000, Mexico City 04510, Mexico

**Keywords:** NFAT5, diabetic retinopathy, neutrophils, inflammatory markers, hyperosmolarity

## Abstract

Diabetic retinopathy (DR) is a major microvascular complication of type 2 diabetes (T2D) and is strongly associated with chronic inflammation. Neutrophils contribute to this inflammatory milieu, and the hyperosmolar stress-responsive transcription factor NFAT5 and its downstream effector aldose reductase (AR) may play crucial roles in this process. NFAT5 regulates AR, which converts glucose to sorbitol; excessive sorbitol accumulation promotes endothelial and retinal cell damage. Given the links between NFAT5, metabolic stress and immune activation, dysregulation of the NFAT5–AR axis in neutrophils may contribute to DR pathophysiology. This study evaluated NFAT5 and AR expression in peripheral blood neutrophils from 150 individuals classified as nondiabetic (*n* = 50), T2D without DR (*n* = 50), or T2D with DR (*n* = 50). Clinical, metabolic, and ophthalmic assessments were performed, and neutrophils were isolated to quantify NFAT5 and AR via ELISA. Associations with renal function, plasma osmolarity (pOSM), and hematological inflammatory ratios (NLR, NMR, NPAR, and SII) were analyzed. T2D-DR subjects presented impaired renal parameters, increased pOSM, reduced eGFR, and elevated NLR and NPAR. NFAT5 and AR levels were significantly increased in T2D-DR neutrophils and correlated positively with pOSM and the inflammatory ratios, whereas NFAT5 correlated inversely with the eGFR. These findings suggest that activation of the NFAT5–AR pathway contributes to neutrophil-driven inflammatory and hyperosmolar dysregulation in T2D and may influence DR progression.

## 1. Introduction

Diabetic retinopathy (DR) is a major microvascular complication of type 2 diabetes (T2D) and remains a leading cause of blindness globally, including in regions such as Mexico [1]. T2D is fundamentally characterized by a combination of insulin resistance, the presence of metabolic syndrome, and a persistent state of chronic hyperglycemia [2]. Chronic hyperglycemia also promotes the formation of reactive carbonyl species such as methylglyoxal, which drives advanced glycation end-product (AGE) formation and protein dysfunction, contributing to insulin resistance, vascular damage, and diabetic complication [3]. The onset and progression of DR are driven by multiple interconnected risk factors that occur against the backdrop of an exacerbated inflammatory environment within the eye. Key systemic factors contributing to this progression include the duration of T2D, sustained hyperglycemia, hypertension and hypertriglyceridemia [4,5]. Recently, we reported that hyperglycemia and hyperosmolarity are biochemical factors associated with an exacerbated spontaneous release of neutrophil extracellular traps (NETs), a chronic inflammatory mechanism of neutrophils observed in subjects with DR [6]. Neutrophils are peripheral inflammatory cells that can be activated by diverse inflammatory stimuli, including danger-associated tissue damage signals, cytokines, and chemokines [7]. Recent studies have shown that neutrophils from diabetic individuals exhibit a different inflammatory response to high-glucose stimulation than neutrophils from nondiabetic subjects [8]. Neutrophils and their inflammatory mechanisms are closely associated with exacerbated tissue inflammation and damage, contributing to a worse prognosis in patients with T2D complications such as diabetic foot ulcers and nephropathy [9,10,11]. In this regard, several hematological indices are used clinically to facilitate the diagnosis and prognosis of the severity of systemic inflammatory diseases, such as COVID-19 [11,12] and endometriosis [13]. Some of these indices include the neutrophil/lymphocyte ratio (NLR), platelet/lymphocyte ratio (PLR), systemic immune inflammation index (SIII), and neutrophil/protein albumin ratio (NPAR), integrating both neutrophil burden and systemic inflammatory/nutritional status through albumin levels, which may better reflect chronic inflammatory states. Recently, NPAR has gained importance in predicting the severity and mortality of diseases such as hepatitis B virus (HBV)-related liver failure [14], metabolic dysfunction associated with hepatic steatosis [15] and cancer [16]. In this sense, neutrophils and their inflammatory indices could have an important function as systemic inflammatory markers of diabetes and its complications.

The nuclear factor of activated T cells 5 (NFAT5), also known as TonEBP [17,18], is a mammalian transcription factor that regulates the gene expression of osmotic regulatory proteins under hyperosmolar conditions [19]. The NFAT5 activation pathway has been described as dependent on multiple signaling pathways, including p38, protein kinase A (PKA), phospholipase C gamma 1 (PLCγ1) and the via of ERK 1/2 after the sensing of osmotic stress signals by aquaporins 1, 2, 4, and 5 (AQP 1, 2, 4, and 5) [20]. NFAT5 transcriptional activity has been shown to increase in response to hyperglycemia, leading to physiological alterations in skeletal muscle in diabetic murine models [21] and promoting angiogenesis and the development of retinopathy in a mouse model [22]; similarly, hyperglycemic conditions can be sensed by AQP1 by human endothelial cells (HAECs) involving the activation of NFAT5, leading to the development of insulin resistance [23].

As part of the polyol pathway, the aldose reductase (AR) enzyme is primarily responsible for converting excess glucose present in diabetes into sorbitol. In this sense, the excess of sorbitol is subsequently metabolized to fructose by the sorbitol dehydrogenase enzyme [24,25,26]. Because of the accumulation of fructose and its metabolites, the formation of glycated proteins and irreversible formation of advanced glycation end products (AGEs) induce cellular stress and tissue damage together with damage to retinal endothelial cells and vascular alterations as complications of diabetes [27,28,29].

Additional studies indicate that NFAT5 contributes to obesity and insulin resistance [30] and that single nucleotide polymorphisms (SNPs) within intronic regions of NFAT5 are associated with T2D susceptibility and its complications [31,32]. Notably, NFAT5 is highly expressed in mesangial and mononuclear cells during diabetic nephropathy [11].

Although the direct role of NFAT5 in immune responses is not fully understood, evidence suggests that the calcineurin–NFAT axis modulates the activation and effector functions of immune cells such as macrophages and neutrophils [33]. Furthermore, other NFAT family members, such as NFAT1, promote the infiltration of activated neutrophils in tumor microenvironments, including those of breast cancer [34].

There is a well-recognized association between the NFAT5–AR axis and the pathophysiology of T2D, suggesting a strong link with the development of vascular complications such as DR. Moreover, NFAT5 may influence the activation of neutrophils, which are key inflammatory cells involved in sustaining and amplifying inflammation in T2D and other chronic diseases.

The purpose of this study was to evaluate the role of the NFAT5–AR pathway in neutrophils from individuals with type 2 diabetes and diabetic retinopathy and to assess its possible contribution to the inflammatory and pathophysiological mechanisms underlying diabetic retinopathy by associating these values with hematological rates useful in clinical prognosis.

## 2. Results

### 2.1. Renal Impairment and Hyperosmolarity Increase with DR and T2D Duration

As shown in Table 1, diabetic subjects presented significant differences in clinical, metabolic, and renal parameters compared with nondiabetic individuals. Metabolic variables, including fasting glucose, %HbA1c, cholesterol, and triglycerides, were significantly altered in both the T2D and T2D-DR groups relative to those in the nondiabetic group.

#### 2.1.1. Renal Function Markers and Plasma Osmolarity Demonstrate Diabetes- and DR-Related Deterioration

**Figure 1:**
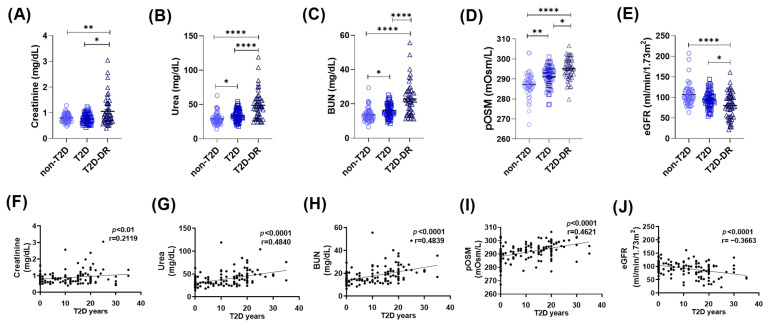
Renal function markers and plasma osmolarity are altered in diabetic subjects and are correlated with T2D duration. (**A**–**E**) Comparison of renal and osmotic parameters among non-T2D, T2D, and T2D-DR subjects. (**A**) Creatinine, (**B**) urea, (**C**) blood urea nitrogen (BUN), (**D**) estimated glomerular filtration rate (eGFR), and (**E**) pOSM. Compared with non-T2D subjects, the T2D and T2D-DR groups presented significantly increased creatinine, urea, BUN, and pOSM levels, along with a reduced eGFR in the T2D-DR group. Significant differences in several parameters were also observed between the T2D and T2D-DR groups. (**F**–**J**) Correlation analyses between years since T2D diagnosis and renal or osmotic markers: (**F**) creatinine, (**G**) urea, (**H**) BUN, (**I**) eGFR, and (**J**) pOSM. Positive correlations were found for creatinine, urea, BUN, and pOSM, whereas the eGFR was significantly negatively correlated with T2D duration. Statistical comparisons were performed via the Kruskal–Wallis and Spearman tests; * *p* < 0.05, ** *p* < 0.01, **** *p* < 0.0001.

To determine whether renal function and osmotic balance differ among the study groups, we evaluated creatinine, urea, blood urea nitrogen (BUN), estimated glomerular filtration rate (eGFR), and plasma osmolarity (pOSM) in nondiabetic (non-T2D), diabetic without retinopathy (T2D), and diabetic retinopathy (T2D-DR) subjects (Figure 1A–E). Creatinine levels were significantly higher in T2D subjects than in non-T2D controls (*p* < 0.05) and further elevated in T2D-DR subjects (*p* < 0.01 vs. non-T2D; *p* < 0.05 vs. T2D) (Figure 1A). Similarly, urea concentrations increased stepwise across groups, with significant differences between non-T2D and T2D patients (*p* < 0.05) and highly significant increases in T2D-DR patients compared with both groups (*p* < 0.0001) (Figure 1B). BUN displayed the same trend, with higher levels in T2D patients than in non-T2D patients (*p* < 0.05) and markedly elevated levels in T2D-DR patients than in both groups (*p* < 0.0001) (Figure 1C). The plasma osmolarity increases with diabetes progression. Compared with that in non-T2D individuals, plasma osmolarity in both T2D and T2D-DR patients was significantly greater (*p* < 0.01 and *p* < 0.0001, respectively). Notably, pOSM was also significantly greater in T2D-DR patients than in T2D patients (*p* < 0.05), indicating worsening osmotic imbalance with retinopathy severity (Figure 1D). eGFR decreases significantly in T2D-DR patients. Consistent with declining renal function, eGFR values were significantly lower in T2D-DR patients than in non-T2D (*p* < 0.0001) and T2D (*p* < 0.05) patients (Figure 1E). These changes reflect the expected deterioration of glomerular filtration associated with long-term diabetes and retinopathy.

#### 2.1.2. Renal Dysfunction and Hyperosmolarity Correlate with the Duration of Type 2 Diabetes

To explore the relationship between disease progression and renal deterioration, we analyzed correlations between years since T2D diagnosis and renal/metabolic variables (Figure 1F–J). Creatinine (Figure 1F) and urea (Figure 1G) were significantly positively correlated with T2D duration (r = 0.2119, *p* < 0.01; r = 0.4840, *p* < 0.0001, respectively). BUN exhibited a similar strong positive association (r = 0.4839, *p* < 0.0001) (Figure 1H). In contrast, the eGFR demonstrated a significant negative correlation with T2D duration (r = −0.3663, *p* < 0.0001), which was consistent with the progressive decline in kidney function over time (Figure 1I). The plasma osmolarity also correlated positively with disease duration (r = 0.4621, *p* < 0.0001), suggesting that cumulative osmotic stress is associated with prolonged hyperglycemia (Figure 1J).

Overall, our findings demonstrate that renal impairment and hyperosmolarity are early and progressively worsening features of T2D, becoming more pronounced in individuals with DR. This dysregulation of renal parameters contributes to a hyperosmolar milieu that may increase cellular stress and amplify inflammatory processes. The strong correlations with T2D duration further support the notion that chronic metabolic dysregulation drives both renal deterioration and osmotic imbalance in the progression of T2D and DR.

### 2.2. Some Systemic Inflammatory Ratios Are Elevated in T2D Patients and Further Increased in T2D-DR Patients

Hematological inflammatory scores have recently gained prominence as accessible biomarkers and prognostic tools in several inflammatory conditions [18,19,20]. In this context, we evaluated whether hematological inflammatory indices are associated with the duration and progression of T2D in the present cohort.

#### 2.2.1. Differential Hematological Inflammatory Indices Across the Non-T2D, T2D, and T2D-DR Groups

**Figure 2:**
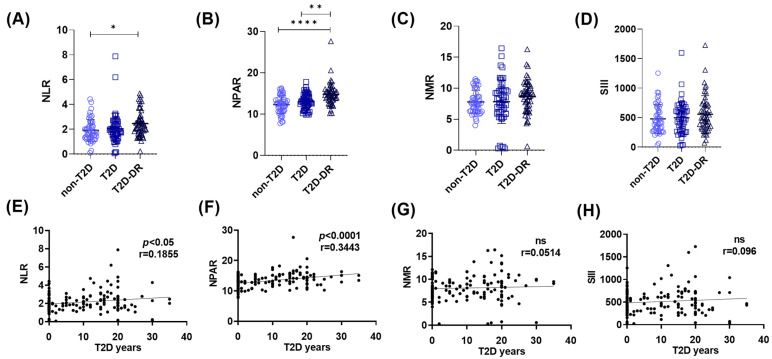
Hematological inflammatory indices across groups and their associations with the duration of T2D. (**A**–**D**) Comparison of systemic inflammatory ratios among non-T2D, T2D, and T2D-DR subjects. (**A**) NLR was significantly greater in T2D-DR patients than in non-T2D patients (* *p* < 0.05). (**B**) NPAR showed the strongest group differences, with significantly elevated values in T2D-DR patients compared with both T2D patients (** *p* < 0.01) and non-T2D patients (**** *p* < 0.0001). (**C**) NMR did not differ significantly among the groups. (**D**) SII also showed no significant group differences. (**E**–**H**) Correlations of inflammatory indices with years since T2D diagnosis. (**E**) The NLR demonstrated a modest but significant positive correlation with T2D duration (* *p* < 0.05, r = 0.1855). (**F**) The NPAR exhibited a strong positive correlation with T2D duration (r = 0.3443). (**G**) NMR data were not significantly correlated with the duration of T2D (ns, r = 0.0514). (**H**) The SII also displayed no significant correlation (ns, r = 0.096). Statistical comparisons were performed via the Kruskal–Wallis and Spearman tests.

We first compared the inflammatory ratios across the groups. The neutrophil–lymphocyte ratio (NLR) was significantly greater in the T2D-DR group than in the non-T2D group (* *p* < 0.05) (Figure 2A). More prominently, the neutrophil percentage–albumin ratio (NPAR) showed robust group differences, with significantly elevated values in T2D-DR patients relative to both T2D patients (** *p* < 0.01) and non-T2D controls (**** *p* < 0.0001) (Figure 2B). In contrast, no significant differences in the neutrophil–monocyte ratio (NMR) or systemic immune-inflammation index (SII) were detected between the groups (Figure 2C,D).

#### 2.2.2. Correlation of Inflammatory Indices with Duration of Type 2 Diabetes

Regarding the duration of T2D, both the neutrophil–lymphocyte ratio (NLR) (* *p* < 0.05, r = 0.1855) and the NPAR (**** *p* < 0.0001, r = 0.3443) were significantly positively correlated with the number of years since T2D diagnosis (Figure 2E,F). In contrast, neither the NMR nor the SII exhibited meaningful correlations with T2D duration (Figure 2G,H). These findings indicate that among the evaluated indices, the NPAR and NLR most closely reflect the inflammatory burden associated with long-term T2D and the presence of diabetic retinopathy.

### 2.3. NFAT5 and AR Levels Correlate with pOSM and the eGFR, Linking Osmotic Stress to Inflammation in T2D-DR

Neutrophils are inflammatory cells with multiple mechanisms implicated in exacerbating and perpetuating inflammation in T2D. We previously reported that neutrophils and their NETosis process, in conjunction with hyperosmolarity conditions, are risk factors for T2D-DR [6]. To determine the role of the hypertonic transcription factor NFAT5 in T2D and DR, we analyzed NFAT5 in peripheral isolated neutrophils from T2D, T2D-DR and non-T2D subjects.

**Figure 3:**
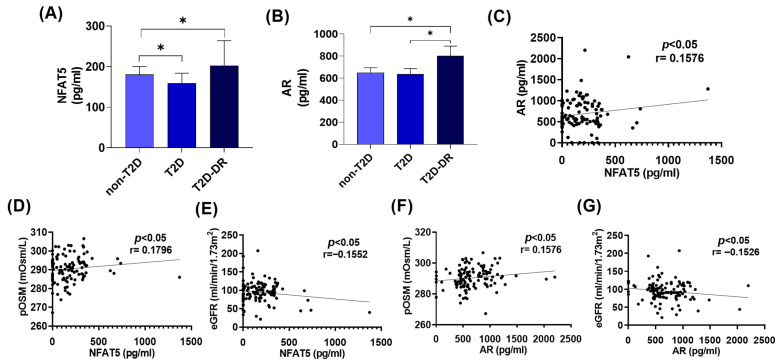
NFAT5 and AR expression in peripheral neutrophils and their associations with osmolar and renal parameters in T2D and T2D-DR patients. (**A**,**B**) Concentrations of NFAT5 and AR in peripheral neutrophils from non-T2D, T2D, and T2D-DR patients. (**A**) NFAT5 levels were significantly different across groups, with a modest decrease in T2D patients compared with non-T2D patients (* *p* < 0.05) and with and increasing NFAT5 values between non-T2D and T2D-DR patients. The bars represent the means ± SEMs. (**B**) AR levels were significantly greater in the T2D-DR group than in both the non-T2D and T2D groups (* *p* < 0.05). (**C**) NFAT5 and AR levels were positively correlated in peripheral isolated neutrophils (* *p* < 0.05, r = 0.1576). (**D**,**E**) Associations between NFAT5 and systemic parameters. (**D**) NFAT5 showed a modest positive correlation with pOSM (* *p* < 0.05, r = 0.1796). (**E**) NFAT5 expression was significantly inversely correlated with eGFR (* *p* < 0.05, r = −0.1552). (**F**,**G**) Associations between AR and systemic parameters. (**F**) AR was positively correlated with pOSM (* *p* < 0.05, r = 0.1576). (**G**) AR was negatively correlated with the eGFR (* *p* < 0.05, r = −0.1526). Statistical comparisons were performed via the Kruskal–Wallis and Spearman tests.

NFAT5 levels were significantly greater in the T2D-DR group than in the non-T2D group (* *p* < 0.05), whereas NFAT5 levels were lower in isolated neutrophils from T2D patients than in those from non-T2D patients (Figure 3A), indicating an association between NFAT5 activation and the inflammatory milieu characteristic of DR. Because NFAT5 directly regulates AR, a key enzyme of the polyol pathway implicated in hyperosmotic stress, we next evaluated AR expression in isolated neutrophils. AR levels were markedly elevated in the T2D-DR group compared with both the T2D group and the non-T2D group (* *p* < 0.05) (Figure 3B). A significant positive correlation was observed between NFAT5 and AR expression (* *p* < 0.05, r = 0.1576) (Figure 3C), which reinforces the coordinated activation of the NFAT5–AR axis in T2D-related inflammation. Moreover, both NFAT5 and AR concentrations were significantly positively correlated with plasma osmolarity (NFAT5: * *p* < 0.05, r = 0.1796; AR: * *p* < 0.05, r = 0.1576) and inversely correlated with eGFR (NFAT5: * *p* < 0.05, r = −0.1552; AR: * *p* < 0.05, r = −0.1526) (Figure 3D–G). Therefore, NFAT5 and AR may underlie the hyperosmolar imbalance in T2D and DR and enhance the associated inflammatory response.

### 2.4. NFAT5 and AR Correlate with Hematological Inflammatory Markers

**Figure 4:**
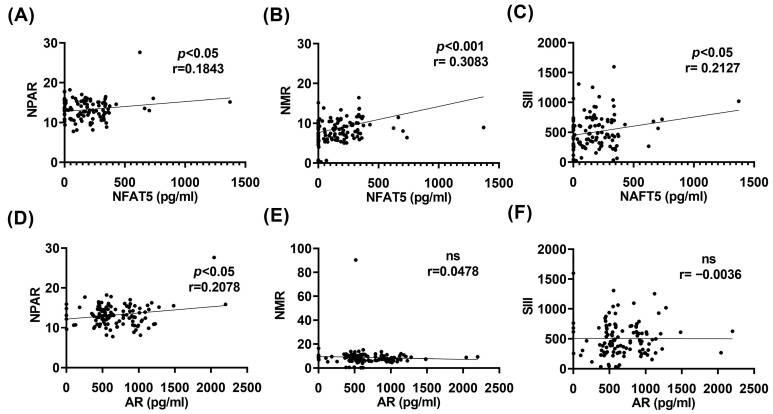
Correlations between NFAT5 or aldose reductase (AR) levels in neutrophils and hematological inflammatory indices. (**A**–**C**) Associations between NFAT5 and inflammatory markers. (**A**) NFAT5 expression was significantly positively correlated with the NPAR (r = 0.1843). (**B**) NFAT5 exhibited a strong positive correlation with NMR (r = 0.3083). (**C**) NFAT5 was also positively correlated with the SII (r = 0.2127). (**D**–**F**) Associations between AR and inflammatory markers. (**D**) AR was significantly positively correlated with the NPAR (r = 0.2078). (**E**) AR did not correlate with the NMR data (ns, r = 0.0478). (**F**) AR was not correlated with the SII (ns, r = −0.0036). Statistical associations were performed via Spearman tests.

Given our previous findings showing altered hematological inflammatory indices across study groups, we next assessed whether NFAT5 and AR levels were associated with these markers. NFAT5 concentrations were significantly positively correlated with the NLR (r = 0.1843, * *p* < 0.05), NPAR (r = 0.3083, * *p* < 0.05), and SII (r = 0.2170, * *p* < 0.05) (Figure 4A–C). Similarly, AR levels were significantly correlated with the NLR (r = 0.2078, * *p* < 0.05), whereas no significant associations were observed with the NPAR or the SII (Figure 4D–F). Taken together, these findings suggest that the NFAT5–AR axis may contribute to the proinflammatory environment characteristic of T2D-DR, in parallel with the renal and hyperosmolar abnormalities observed in T2D. Importantly, the associations of NFAT5 and AR with multiple hematological inflammatory markers support their potential involvement in neutrophil activation and systemic inflammatory dysregulation.

## 3. Discussion

Hyperosmolar and hyperglycemic conditions are hallmarks of diabetes. These metabolic disturbances predispose individuals to significant physiological alterations that amplify inflammation and tissue injury [35,36]. Multiple factors associated with cellular stress and metabolic imbalance exacerbate inflammatory responses, including chronic hyperglycemia and, more recently, hyperosmolarity. In our previous study [6], we reported that clinical markers of uncontrolled glycemia—such as glycated hemoglobin—and indicators of renal dysfunction that increase plasma osmolarity, together with inflammatory mediators and neutrophil-derived NETs, collectively contribute to the risk and severity of DR. Consistent with this, in the present study, we observed clear alterations in renal function among T2D and T2D-DR patients, including elevated levels of creatinine, urea, and BUN, as well as increased plasma osmolarity and a significant reduction in the eGFR. These parameters were positively correlated with the duration of T2D. Our findings align with previous reports that abnormal renal indices correlate with poor glycemic control in T2D patients [37], which was also evident in our cohort through increased %HbA1c and FG.

Hematological inflammatory indices—derived from blood cell proportions—are increasingly used as prognostic markers in various conditions, including COVID-19, cancer, preeclampsia, and endometriosis [13,14,15,35,36]. Accordingly, in the present study, the NLR and NPAR were elevated in subjects with DR and were positively associated with the duration of T2D. These results are supported by evidence showing that abnormal hematological ratios correlate with poor glycemic control [37,38] and that microvascular complications of diabetes may also be linked to parameters such as hematocrit, the MPV, the NLR, and the PLR [39]. Thus, our findings highlight the association of inflammation with homeostatic disruption in T2D and DR.

Given that neutrophil-related indices such as the NLR, NPAR, and NMR were altered in our patients—and that many T2D and T2D-DR patients exhibited hyperosmolar states—we investigated the behavior of the NFAT5–AR axis in neutrophils. Notably, the hypertonicity-responsive transcription factor NFAT5 was elevated in neutrophils from T2D-DR subjects compared with those from non-T2D subjects. Similarly, AR expression was greater in the T2D-DR group than in the other groups, and the two proteins were significantly positively correlated. Both NFAT5 and AR correlated positively with plasma osmolarity and inversely with eGFR. NFAT5 is tightly linked to inflammation in dia-betes because of its central role in hyperosmotic stress adaptation, and hyperosmolarity itself is increasingly recognized as a driver of vascular complications in DR [20]. In support of this, elevated NFAT5 expression has been associated with the severity of inflammatory processes, such as lung injury, in T2D mouse models [40] and with CD8^+^ T-cell exhaustion in tumor contexts [41]. The known association of diabetic kidney disease with an increased risk of DR progression further reinforces the connection among hyperosmolar stress, NFAT5 regulation, and vascular dysfunction [42,43].

Although diabetes is an inflammatory condition, unexpectedly, the NFAT5 factor showed a decrease in subjects with type 2 diabetes compared to nondiabetic subjects. Interestingly, it has been reported that healthy subjects exhibit increased expression of NFAT5 in dermal biopsies, contrary to what occurs in subjects with type 1 diabetes (T1D), which may explain the osmotic imbalance present in diabetes [44]. In the same way, interestingly, the NFAT5 factor has been found to be regulated by several signaling pathways, including STAT3, protein kinase A (PKA), protein kinase C (PKC) and extracellular signal-regulated kinases (ERK) [40]. In a model of pulmonary tuberculosis (TB) associated with type 2 diabetes, NFAT5 overexpression was observed to be associated with TB severity and exacerbated inflammation; however, disease severity is reduced by the transcription of miR-19b/1281 [45]. In this case, a similar suppression mechanism may exist in diabetic subjects, where NFAT5 expression is regulated overcontrol, while in subjects who also have diabetic retinopathy (DR), an imbalance may exist that prevents the regulation of NFAT5 expression, reflecting a stage-dependent NFAT5 dynamics rather than contradictory results.

AR, the rate-limiting enzyme of the polyol pathway, is upregulated in hyperglycemic conditions and converts excess glucose into sorbitol. In diabetes, increased sorbitol contributes to oxidative stress and vascular damage [26,46]. Our results suggest that elevated AR expression reflects increased inflammatory activity, as previously documented under hyperglycemic stress [27]. The concurrent increase in NFAT5 and AR suggests coordinated or possibly synergistic activation of osmotic and inflammatory pathways in T2D and T2D-DR [27,47,48].

NFAT5 regulates inflammatory functions in monocytes, macrophages, astrocytes, microglia, and T cells [20]. Neutrophils—potent mediators of inflammation—contribute to insulin resistance, exaggerated responses to hyperglycemia, and tissue damage underlying diabetic vascular complications [49,50]. Accordingly, in the present study, NFAT5 and AR were positively correlated with inflammatory hematological ratios, such as the NPAR and NMR, and NFAT5 was additionally correlated with SIII. These associations support the hypothesis that the NFAT5–AR pathway could modulate neutrophil inflammatory behavior in T2D and DR contexts. Supporting evidence indicates that NFAT5 and AR play roles in microglial activation [51] and promote neuroinflammation after ischemic stroke [52]. NFAT5 expression in macrophages has also been linked to migration and pathological activity during kidney injury [53,54]. Therefore, NFAT5 may contribute broadly to the inflammatory responses of innate immune cells in diabetes while also shaping vascular complications such as nephropathy and retinopathy.

It is important to consider the emerging role of neutrophils in the pathophysiology of diabetic retinopathy. In the context of the NFAT5–AR axis, increasing evidence suggests that neutrophils contribute to retinal microvascular dysfunction and the exacerbation of tissue damage. Neutrophils can be activated by damage-associated molecular patterns (DAMPs), including glycated proteins and oxidative products such as advanced glycation end products (AGEs). Under diabetic hyperglycemic conditions, these damage signals, together with the hyperosmolar milieu, may promote neutrophil activation and inflammatory responses involving the NFAT5–AR pathway, potentially enhancing endothelial adhesion, reactive oxygen species production, and neutrophil extracellular trap formation. Although the present study does not directly assess neutrophil activation or function, the observed associations are consistent with a broader inflammatory environment in which neutrophils may play a contributory role. Future mechanistic and functional studies will be required to directly evaluate neutrophil involvement and to delineate their interaction with the molecular pathways identified in this work.

This comparative, correlational study examined NFAT5 and aldose reductase (AR) expression in neutrophils from subjects with type 2 diabetes and diabetic retinopathy and analyzed their associations with clinical variables related to renal function and systemic inflammatory status. Given the complexity of immune–metabolic regulation, bidirectional effects of NFAT5 signaling are plausible, as its activation is highly context dependent and influenced by osmotic thresholds, inflammatory cues, and microenvironmental conditions [28]. Due to limitations in sample availability, additional functional assays could not be performed. Future prospective studies incorporating functional approaches will therefore be required to elucidate the mechanistic role of the NFAT5–AR axis in neutrophil activation and its contribution to disease pathophysiology.

In summary, our findings support the involvement of the NFAT5–AR axis in neutrophils as a potential mediator of hyperosmolar and inflammatory dysregulation in T2D and DR. However, additional functional and mechanistic studies are needed to clarify its precise contribution to disease progression and to evaluate its value as a biomarker or therapeutic target.

## 4. Materials and Methods

### 4.1. Reagents

Phosphate-buffer solution (PBS, pH 7.2), Hank’s balanced salt solution (HBSS), and Trypan blue were purchased from Sigma–Aldrich (Saint Louis, MI, USA). Bovine serum albumin (BSA) was obtained from Calbiochem (San Diego, CA, USA). Polymorphoprep was purchased from Alere Technologies AS (Jena, Germany). Plastic tubes were purchased from Corning, Inc. (Corning, NY, USA). NFAT5 and AR ELISA kits were purchased from Myo BioSource (San Diego, CA, USA). The DC protein assay kit II was purchased from Bio-Rad (Hercules, CA, USA).

### 4.2. Study Groups

Diabetic subjects of both genders, aged ≥18 years, with or without a diagnosis of diabetic retinopathy, were recruited at the Institute of Ophthalmology “Fundación Conde de Valenciana” in Mexico City between March 2024 and May 2025. Nondiabetic individuals were also recruited as controls. All procedures were conducted in accordance with the Institutional ERB guidelines of the Institute of Ophthalmology “Fundación Conde de Valenciana” (CEI-2023/06/01). A total of 150 participants were enrolled and allocated into three groups: diabetic without retinopathy (T2D; *n* = 50), diabetic with retinopathy (T2D-DR; *n* = 50), and nondiabetic control groups (non-T2D; *n* = 50). Diabetic subjects were defined as individuals with HbA1c ≥ 6.5%, whereas nondiabetic controls presented HbA1c ≤ 5.6%. Subjects with other conditions—including autoimmune diseases, renal disease, recent surgeries, or active infections—were excluded from the study. All enrolled participants underwent a complete clinical and ophthalmological evaluation, along with biochemical testing, including urinalysis, complete blood count, and blood chemistry analysis.

### 4.3. Renal Function and Inflammatory Indices Calculation

Renal function, expressed as the estimated glomerular filtration rate (eGFR), was calculated via the MDRD-6 (Modification of Diet in Renal Disease) equation. The plasma osmolarity (pOsm) was determined via the following formula:

mOsm/L = 2(Na + K) + (Glucose/18) + (BUN/2.8)(1)

Plasma osmolarity reflects the serum concentration of osmotically active solutes, primarily sodium (Na), potassium (K), glucose and blood urea nitrogen (BUN).

Hematological inflammatory indices were calculated as follows: neutrophil-to-lymphocyte ratio (NLR) = neutrophil absolute count/lymphocyte absolute count; neutrophil-to-monocyte ratio (NMR) = neutrophil absolute count/monocyte absolute count; neutrophil percentage-to-albumin ratio (NPAR) = neutrophil percentage/serum albumin concentration; and systemic immune-inflammation index (SIII) = (absolute neutrophil count × absolute platelet count)/absolute lymphocyte count.

### 4.4. Polymorphonuclear Cell Isolation

Twenty milliliters of peripheral blood was collected from recruited subjects on heparin vacutainer tubes within 6–8 h of fasting. The blood samples were processed immediately for neutrophil isolation as follows.

As previously described [5], human neutrophils were isolated via a density gradient method in which whole blood was placed on an equal volume of polymorphoprep solution and then centrifuged at 500× *g* for 35 min at RT°C without breaking or accelerating. Then, the polymorphonuclear rings were collected and washed with 0.1 M (0.1 M) ice-cold buffered PBS and centrifuged at 500× *g* for 8 min at 4 °C. The remaining erythrocytes were lysed with lysis solution for 10 min at 4 °C [NH_4_Cl 152.7 mM, Na_2_EDTA 0.1 mM, NaHCO_3_ 9.0 mM at pH 7.4], and the total number of cells and viability were tested via the trypan blue exclusion test.

### 4.5. Total Neutrophil Protein Extraction

Two million purified peripheral neutrophils were placed in a tube with 100 μL of RIPA buffer [25 mM Tris-HCl, pH 7.6; 150 mM NaCl; 1% Triton X-100; 1% sodium deoxycholate; 0.1% SDS; 1% sodium orthovanadate; and protease inhibitors], and vigorous homogenization was performed, taking care not to create foam in the samples. The samples were immediately frozen at −70 °C and stored until protein quantification.

### 4.6. Protein Quantification

The quantification of proteins was performed according to the manufacturer’s instructions. Briefly, the samples were thawed at room temperature (RT) and centrifuged at 18,000× *g* for 30 min at 4 °C. The pellet was discarded, and the supernatant was transferred to a new tube. A standard curve (2–10 µg/µL) was generated using a 1 mg/mL BSA standard. Five microliters of each sample was placed on a 96-well plate together with the standard curve; then, 25 µL of reagent A + S solution was added to the samples and standards. After homogenization, 200 µL of reagent B was added to the samples and standards, which were subsequently incubated for 15 min in darkness. The plate was immediately read with a spectrophotometer at 750 nm.

### 4.7. ELISA of NFAT5 and Aldose Reductase

The assays were performed according to the manufacturer’s instructions as follows. Briefly, all reagents were thawed for at least 30 min before use. Samples and standard curves were generated following the manufacturer’s instructions, considering a final concentration of 20 µg of protein for the neutrophil extracts. One hundred microliters of sample with 20 µg of total protein or standard were placed on a 96-well plate and incubated at 37 °C for 90 min. After washing, the plate was incubated for 60 min at 37 °C with 100 µL of working solution of biotin-labeled antibody. The plate was subsequently washed and incubated for 30 min at 37 °C with 100 µL of a working solution of HRP-conjugated streptavidin. Finally, the plate was incubated for 20 min at 37 °C in the dark with a chromogenic TMB solution. The colorimetric reaction was stopped, and the plate was immediately read at 450 nm in a microplate spectrophotometer. The protein concentration was calculated via interpolation of the standard curve via the software CurveExpert 1.4 Hyams Development (Malibu, CA, USA). The data are expressed as the means in pg/mL ± SE.

### 4.8. Statistical Analysis

The data are expressed as the means ± SEs. Normality was assessed prior to the statistical analyses. Because several variables did not meet normality assumptions, nonparametric tests were used. Group comparisons were performed via the Kruskal–Wallis test followed by Dunn’s post hoc test, with *p* < 0.05 considered statistically significant. Linear associations between variables were evaluated via a one-tailed nonparametric Spearman correlation test with a 95% confidence interval, considering the hypothesis that higher NFAT5/AR levels will be associated with renal dysfunction and inflammatory parameters, where r values of −1, 0, and +1 indicate perfect negative correlation, no correlation, and perfect positive correlation, respectively.

All statistical analyses and graphs were generated via GraphPad Prism 9 (GraphPad Software, La Jolla, CA, USA).

## 5. Conclusions

This study revealed that renal impairment, systemic hyperosmolarity, and altered hematological inflammatory indices are progressively aggravated across the spectrum from T2D to T2D-DR. These systemic alterations are associated with increased expression of NFAT5 and its downstream effector, AR, in peripheral neutrophils. The coordinated activation of the NFAT5–AR axis, together with its correlations with plasma osmolarity, eGFR, and inflammatory ratios, suggests that this pathway functions as a molecular integrator of metabolic, osmotic, and inflammatory stress in T2D-DR. Our findings support a model in which chronic hyperglycemia and renal decline generate a hyperosmolar environment that enhances neutrophil activation through NFAT5-dependent mechanisms, thereby contributing to the inflammatory cascade underlying diabetic retinopathy. The associations between NFAT5–AR activation and systemic inflammatory markers further highlight their potential utility as accessible biomarkers of disease severity. Overall, the NFAT5–AR axis has emerged as a promising target for future therapeutic interventions aimed at mitigating inflammation and microvascular damage in T2D-DR patients. Longitudinal and mechanistic studies will be essential to determine whether the modulation of this pathway can attenuate neutrophil-driven inflammation and influence the progression of diabetic retinopathy.

## Figures and Tables

**Table 1:** Demographic, clinical, metabolic and renal parameters of study subjects.

Subjects (*n* = 150)	Non-T2D (*n* = 50)	T2D (*n* = 50)	T2D-DR (*n* = 50)	*p* Value
Age (yo)	45.6 (±11.0)	60.0 (±10.5)	59.4 (±11.0)	* *p* < 0.0001 ^£^ *p* < 0.001
Gender (F/M)	27/23	30/20	26/22	ns
BMI	27.6 (±5.1)	29.3 (±5.8)	27.2 (±4.5)	ns
Time T2D (years)	--	11.0 (±8.2)	17.5 (±6.5)	^&^ *p* < 0.0001
HBP (yes/no)	4/46	14/36	25/25	ns
SBP (mmHg)	118.9 (±14.9)	125.5 (±26.5)	125.2 (±25.3)	* *p* = 0.0221 ^£^ *p* = 0.0151
DBP (mmHg)	74.3 (±9.7)	74.5 (±16.0)	76.2 (±15.4)	ns
FG (mg/dL)	88.8 (±7.7)	139.1 (±48.8)	173.1 (75.3)	* *p* < 0.0001 ^£^ *p* < 0.001
HbA1b (%)	5.4 (±0.3)	7.7 (±1.2)	9.1 (±2.6)	* *p* < 0.0001 ^£^ *p* < 0.001
Cholesterol (mg/dL)	183.6 (±32.7)	179.3 (±44.1)	192.3 (±54.5)	ns
HDL (mg/dL)	52.6 (±10.0)	49.2 (±11.0)	48.6 (±12.1)	* *p* = 0.0368 ^£^ *p* = 0.0251
LDL (mg/dL)	106.8 (±24.6)	104 (±36.6)	117.8 (±47.3)	ns
Triglycerides (mg/dL)	135.4 (±63.8)	156.7 (±82.6)	173.1 (±147.7)	* *p* = 0.0373 ^£^ *p* = 0.0083
Drug use				
Insulin (Y/N)	0/50	19/31	25/25	ns
Metformin (Y/N)	0/50	39/21	32/28	ns
Other hypoglycemic agents (Y/N)	0/50	25/25	24/26	ns
Hypolipidemic agents (Y/N)	3/47	14/36	14/36	ns

Non-T2D, nondiabetic controls; T2D, type 2 diabetic subjects; T2D-DR, type 2 diabetic subjects with retinopathy (DR); yo, years old; F/M, female/male; BMI, body mass index; HBP, high blood pressure; SBP, systolic blood pressure; DBP, diastolic blood pressure; FG, fasting glucose; HbA1b, glycated hemoglobin; HDL, high-density lipoprotein cholesterol; LDL, low-density lipoprotein cholesterol; eGFR, estimated glomerular filtration rate; pOsm, plasma osmolarity; * *p* non-T2D vs. T2D; ^£^ *p* non-T2D vs. T2D-DR; ^&^ *p* T2D vs. T2D-DR; ns, nonsignificant difference. Y/N, yes, no. Other hypoglycemic agents include mainly dapagliflozin and glibenclamide. Hypolipidemic are mainly atorvastatin and bezafibrate.

## Data Availability

The original contributions presented in this study are included in the article. Further inquiries can be directed at the corresponding author. Data will be shared upon reasonable requests.
